# Pulmonary tuberculosis and non-recent immigrants in Japan – some issues for post-entry interventions

**DOI:** 10.5365/wpsar.2017.8.3.003

**Published:** 2017-12-15

**Authors:** Lisa Kawatsu, Kazuhiro Uchimura, Akihiro Ohkado, Seiya Kato

**Affiliations:** a Department of Epidemiology and Clinical Research, the Research Institute of Tuberculosis, Japan Anti-Tuberculosis Association, Japan.; b Nagasaki University Graduate School of Biomedical Sciences, Nagasaki, Japan.; c The Research Institute of Tuberculosis, Japan Anti-Tuberculosis Association, Japan.

## Abstract

Foreign-born persons are considered one of the high-risk populations for tuberculosis (TB), and numerous studies have discussed the potential role of pre-entry TB screening for immigrants. However, rates of TB disease among immigrants can remain high several years after entry. In Japan, approximately 50% of TB among foreign-born persons occurs among those who have entered Japan more than five years before being diagnosed, i.e. non-recent immigrants. However, little attention has been paid so far to the issue of TB control among the non-recent immigrants. A detailed analysis of the Japan Tuberculosis Surveillance data was therefore conducted to describe the characteristics of TB among non-recent immigrants and discuss policy implications in terms of post-entry interventions in Japan. The main findings were as follows: 1) the proportion of pulmonary TB cases aged 65 years and older was higher among non-recent than recent immigrants (9.8% vs 1.2%); 2) the proportion of those with social risk factors including homelessness and and being on social welfare assistance was higher among non-recent than recent immigrants; and 3) the proportion of those detected via routine screening at school or workplace was significantly lower among non-recent immigrants aged between 25 and 64 than among recent immigrants in the same age group (15.4% vs 28.7%). Our results suggested the need to increase the opportunities for and simultaneously improve the take-up rate of community-based screening for non-recent immigrants.

## Background

The proportion of tuberculosis (TB) borne by foreign-born persons, especially in low-incidence countries, has been increasing. (*1*) Several of these countries have adopted screening programmes for immigrants that may take place either before entering the destination country, at entry or post entry. (*1*) The effectiveness of these programmes has been discussed elsewhere; (*2*) however, rates of TB disease among immigrants can remain high several years after entry, (*3*, *4*) and return and repeated visits to their country of origin may be a significant risk factor for immigrants as well. (*5*, *6*) Post-entry intervention is, therefore, also potentially important in considering TB control among foreign-born populations.

Japan is a TB medium-burden country with a notification rate of 14.4 per 100 000 population in 2015. (*7*) Although the proportion of foreign-born persons among the total cases is relatively small compared to similarly industrialized countries, between 2005 and 2014, it steadily increased by approximately 1.7 times (from 3.5% to 5.8%) and among those aged 20 to 29 by 2.5 times (from 17.8% to 44.1%). (*7*) It has also been estimated that the TB notification rate per 100 000 population among foreign-born persons increased from 40.7 in 2007 to 56.2 in 2016, in contrast to the decreasing notification rate among the general population. (*8*) Furthermore, data from the Japan TB Surveillance (JTBS) indicate that approximately 50% of TB among foreign-born persons occurs among those who entered Japan more than five years before being diagnosed. (*9*) Although discussions have begun regarding the possible impact of pre-entry TB screening if it were introduced in Japan, little attention has been paid so far to the issue of TB control among these non-recent immigrants. Our objective, therefore, was to describe and analyse the characteristics of TB among non-recent immigrants compared with recent immigrants who had entered Japan within five years of being diagnosed and to discuss policy implications in terms of post-entry interventions in Japan.

### Japan Tuberculosis Surveillance

Japan introduced the first nationwide computerized TB surveillance system, the JTBS, in 1987. TB is a notifiable disease, and public health centres (PHCs) are responsible for collecting and entering the data of notified patients to the system. The data are summarized every month and annually and are made available online. Mechanisms to ensure data quality include the system’s automatic verification programme as well as regular meetings attended by local staff from hospitals and PHCs. Periodic refresher trainings on data entry are also provided to PHC staff across the nation. (*9*)

Data regarding the nationality of patients (either Japanese or non-Japanese) were added to the JTBS in 1998. The country name and the timing of entry (either within five years, more than five years or unknown) were added in 2007. In 2012, the category of nationality was changed to country of birth (either Japan-born or foreign-born); for the foreign-born persons, the name of the country and the year of entry are simultaneously collected.

## Methods

We conducted a cross-sectional study whereby aggregated cohort data of pulmonary TB (PTB) cases newly notified to the JTBS between 1 January 2007 and 31 December 2014 were analysed. A TB case was defined as either a person whose biological specimen was positive by smear, culture or other examinations or a person without bacteriological confirmation but active TB was diagnosed by a clinician. A PTB case includes both those exclusively with pulmonary or bronchial infection and those with concomitant extrapulmonary disease. Immigrants were defined as either those patients whose nationality has been recorded as non-Japanese before 2012 or those whose county of birth was foreign-born after 2012. Those whose nationality or country of birth was recorded as unknown were excluded from the analysis (*n* = 3830, 2007–2014). Non-recent immigrants were defined as those who had entered Japan more than five years before being diagnosed or whose timing of entry was unknown; recent immigrants were those who had entered Japan within five years of being diagnosed. Characteristics of PTB among non-recent immigrants were analysed and compared with the recent immigrants in terms of sex, age groups, countries of birth, job categories, modes of case detection and treatment outcomes. Standardized death rates were calculated using the Japanese population of 1985 as reference. χ^2^ tests or Fisher’s exact tests were conducted to compare proportions, and Bonferrni correction was applied for multiple comparisons. R version 3.1.3 (R Development Core Team, Vienna, Austria) was used for all statistical analyses.

### Ethics

Ethical clearance was deemed not necessary, as the electronic JTBS data do not include case identifiers per the Ethical Guidelines for Epidemiological Research established by the Ministry of Education, Culture, Sports, Science and Technology and Ministry of Health, Labour and Welfare of Japan.

## Results

### General characteristics

**Fig. 1:**
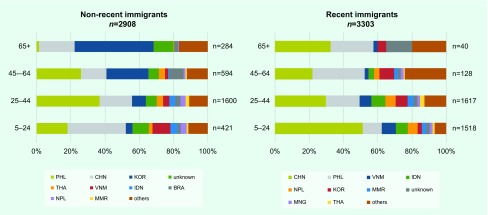
Countries of birth among non-recent and recent immigrants with pulmonary TB, newly notified, by age groups, 2007–2014

Between 2007 and 2014, a cumulative total of 6211 foreign-born PTB cases were notified; 46.8% (*n* = 2908) were non-recent and 53.2% (*n* = 3303) were recent immigrants. General characteristics of the two groups are summarized in **Table 1**. The age distribution was significantly different with a higher proportion of those aged 65 and above among the non-recent than among the recent immigrants (9.8% versus 1.2%). The proportion of females among the non-recent immigrants was significantly greater than that among the recent immigrants (58.0% versus 49.9%, *P* < 0.001). The overall distribution of cases by country of birth was also significantly different. Furthermore, looking at the country of birth by age groups, the recent immigrants from the People’s Republic of China (China) and the Philippines combined contributed approximately 50% of all recent immigrants for all age groups; among the non-recent immigrants, the proportion of those from the Republic of Korea increased with increasing age group. Among those aged 65 and above, those from the Republic of Korea and China contributed approximately 66% of the non-recent immigrants (**Fig. 1**).

**Table 1:** General characteristics of non-recent and recent immigrants with pulmonary TB, newly notified, 2007–2014

**-**	**-**	Non-recent immigrants		Recent immigrants		p-value
		n	%	n	%	
-	**Total**	**2908**	**100**	**3303**	**100**	-
**Age group**	-	-	-	-	-	< 0.001
-	5–14	9	0.3	25	0.8	-
-	15–24	421	14.5	1493	45.2	-
-	25–44	1600	55	1617	49	-
-	45–64	594	20.4	128	3.9	-
-	65+	284	9.8	40	1.2	-
**Sex**	-	-	-	-	-	< 0.001
-	Male	1221	42	1654	50.1	-
-	Female	1687	58	1649	49.9	-
**Country of birth**	-	-	-	-	-	< 0.001
-	China	598	20.6	1301	39.4	-
-	Philippines	827	28.4	544	16.5	-
-	Republic of Korea	424	14.6	165	5	-
-	Indonesia	76	2.6	247	7.5	-
-	Viet Nam	113	3.9	241	7.3	-
-	Nepal	69	2.4	189	5.7	-
-	Others	801	27.5	616	18.6	-
-	Total*	**2194**	**100**	**1745**	**100**	-
**Job category**	-	-	-	-	-	< 0.001
-	Full-time workers#	829	37.8	631	36.2	-
-	Unemployed	497	22.7	293	16.8	-
-	Temporary	297	13.5	256	14.7	-
-	High-school & university/college students	134	6.1	379	21.7	-
-	Homemakers	228	10.4	91	5.2	-
-	Unknown	115	5.2	44	2.5	-
-	Self-employed	65	3	26	1.5	-
-	Others	23	1	10	0.6	-
-	Health-care professionals	6	0.3	15	0.9	-

Non-recent immigrants: immigrants who had entered Japan more than five years before being diagnosed with TB or whose timing of entry was unknown. Recent immigrants: immigrants who had entered Japan within five years before being diagnosed with TB.

* Total number of immigrants with PTB aged 25 to 64 years old

^#^ Full-time employed workers, excluding health-care professionals

Non-recent immigrants: immigrants who had entered Japan more than five years before being diagnosed with TB or whose timing of entry was unknown. Recent immigrants: immigrants who had entered Japan within five years before being diagnosed with TB.

PHL: Philippines, CHN: China, KOR: Republic of Korea, THA: Thailand, VNM: Viet Nam, IDN: Indonesia, BRA: Brazil, MNG: Mongolia, NPL: Nepal, MMR: Myanmar

Job categories of those aged between 25 and 64 years old were also compared. There was a significant difference in the overall distribution of job categories with a higher proportion of those with no regular income, i.e. unemployed and homemaker, and a lower proportion of high-school, university and college students among the non-recent compared with the recent immigrants (33.0% vs 22.0%, 6.1% vs 21.7%) (**Table 1**).

### Case detection of PTB among non-recent immigrants

**Table 2** summarizes the modes of detection of non-recent immigrants with PTB compared with that of recent immigrants among the working-age group (aged 25 to 64) and the elderly (aged 65 and above). In the working-age group, there was a significant difference in the overall distribution of the modes of detection with the proportion of those with TB symptoms detected at medical institutions (64.7% vs 56.2%, *P* < 0.001, with Bonferroni correction) and via health checks as outpatients or inpatients for other diseases (7.6% vs 2.3%, *P* < 0.001, with Bonferroni correction) significantly greater; those detected via routine screening, either at workplace or school, were significantly smaller (15.4% vs 28.7%, *P* < 0.001, with Bonferroni correction) among non-recent than recent immigrants. The odds ratios of non-recent migrants being detected at medical institutions with TB symptoms and via health checks were 1.48 and 3.50, respectively, while the odds ratio of being detected via routine screening was 0.45. No significant difference in the distribution of modes of case detection between non-recent and recent immigrants was observed among those age 65 and above.

**Table 2:** Modes of detection among non-recent and recent immigrants with pulmonary TB, newly notified, by age group, 2007–2014

Mode of detection	Aged 25–64				**-**	Aged 65+				**-**
	Non-recent immigrants		Recent immigrants			Non-recent immigrants		Recent immigrants		
	n	%	n	%	p-value	n	%	n	%	p-value
Total	2194	100.0	1745	100.0	**-**	284	100.0	40	100.0	**-**
-	-	-	-	-	< 0.001	-	-	-	-	0.860
Routine school and workplace screening	338	15.4	503	28.7	-	3	1.1	1	2.5	-
Routine community-based screening	37	1.7	18	1.0	-	4	1.4	1	2.5	-
Individual health check	56	2.6	60	3.4	-	3	1.1	1	2.5	-
Contact investigation	109	5.0	79	4.5	-	3	1.1	0	0.0	-
Other mass investigation	18	0.8	35	2.0	-	1	0.4	0	0.0	-
Hospital visit with TB symptoms	1420	64.7	981	56.2	-	178	62.7	29	72.5	-
Health check as out- or inpatient	166	7.6	40	2.3	-	86	30.3	8	20.0	-
Others	17	0.8	13	0.7	-	5	1.8	0	0.0	-
Unknown	21	1.0	13	0.7	-	1	0.4	0	0.0	-
During TB follow-up	12	0.5	3	0.2	-	0	0.0	0	0.0	-

Non-recent immigrants: those who had entered Japan more than five years before being diagnosed with TB or whose timing of entry was unknown. Recent immigrants: those who had entered Japan within five years before being diagnosed with TB.

### Social risk factors of PTB among non-recent immigrants

**Table 3** summarizes the social risk factors besides unemployment of non-recent immigrants with PTB compared with that of recent immigrants among the working-age group and the elderly. The proportions of those with a history of homelessness (2.1% vs 0.9%, *P* = 0.02) and those receiving social welfare benefits (8.2% vs 1.3%, *P* < 0.001) were significantly greater among non-recent than recent immigrants with PTB; the same tendency was observed for both age categories.

**Table 3:** Social risk factors of non-recent and recent immigrants with pulmonary TB, newly notified, 2007–2014

**-**	**-**	Non-recent immigrants		Recent immigrants		**-**
		n	%	n	%	p-value
All ages	History of homelessness*	24/1170	2.1	6/869	0.9	0.020
	Receiving social welfare benefit*	193/2361	8.2	23/1723	1.3	< 0.001
Aged 25–64	History of homelessness*	18/1028	1.8	6/855	0.7	0.070
	Receiving social welfare benefit*	120/2083	5.8	22/1685	1.3	< 0.001
Aged 65+	History of homelessness*	6/142	4.2	0/14	0.0	NA
	Receiving social welfare benefit*	73/278	26.3	Jan-38	2.6	0.001

Non-recent immigrants: those who had entered Japan more than five years before being diagnosed with TB or whose timing of entry was unknown. Recent immigrants: those who had entered Japan within five years before being diagnosed with TB.

* The denominators do not necessarily equal to the total number of non-recent and recent immigrants due to missing data.

### Treatment outcome of PTB among non-recent immigrants

**Table 4** summarizes the treatment outcomes at the end of 12 months of non-recent immigrants with PTB compared with those of recent immigrants. The proportion of those who had died was significantly higher among the non-recent immigrants (3.2% vs 0.3%, *P* < 0.001, with Bonferroni correction); after adjusting for age, the standardized mortality rate ratio was 2.3.

**Table 4:** Treatment outcomes of non-recent and recent immigrants with pulmonary TB, newly notified, 2007–2014

**-**	Non-recent immigrants		Recent immigrants	
	n	%	n	%
**Total**	**2908**	**100.0**	**3303**	**100.0**
Success	1743	59.9	1857	56.2
Died	93	3.2	10	0.3
Lost to follow-up	215	7.4	242	7.3
Treatment failed	16	0.6	17	0.5
Still on treatment	198	6.8	185	5.6
Unevaluated	366	12.6	404	12.2
Transferred out	277	9.5	588	17.8

Non-recent immigrants: those who had entered Japan more than five years before being diagnosed with TB or whose timing of entry was unknown. Recent immigrants: those who had entered Japan within five years before being diagnosed with TB.

## Discussion

Two distinctive issues in TB among the non-recent immigrants were identified in our study: a larger proportion of those aged 65 and above and a smaller proportion of those being detected via routine school and workplace screening among those aged between 25 and 64.

For the first issue, a significantly greater proportion of non-recent than recent immigrants were aged 65 and above (9.8% vs 1.2%), 66% of whom were from the Republic of Korea and China. Several studies have raised the issue of poor socioeconomic and health status of older foreign-born residents in Japan, especially those from Asia, including higher smoking and drinking rates. (*10*, *11*) It has also been suggested that higher morbidity and mortality among the older foreign-born residents may to a certain extent be attributable to long years of poor working and living conditions in Japan. (*11*)

We also found the proportion of those with social risk factors, such as a history of homelessness and those receiving social welfare benefits, were significantly higher among non-recent than recent immigrants. Among those aged 65 and above, the difference was even more evident with the proportion of those with a homeless history being 4.2% and 0.0% and of those receiving social welfare benefits being 26.3% and 2.6% among the non-recent and recent immigrants, respectively. These socioeconomic factors have been reported to be associated with poor treatment outcomes, including death and prolonged treatment. (*12*)

The second issue concerned the proportion of those detected via routine screening at school or workplace, which was significantly smaller among non-recent than recent immigrants aged between 25 and 64 (15.4% vs 28.7%). In Japan, under the Infectious Diseases Control Law, routine TB screening is mandatory for school students, teachers and employees of selected institutions including hospitals, social welfare facilities and nursing homes for the elderly. For full-time employees aged 40 and above in other industries, chest X-rays are included in the annual workplace health check per the Industrial Safety and Health Law. From the job categories of the JTBS, it is reasonable to assume that that full-time workers; health-care professionals; and high-school, university and college students are eligible for those routine screening. According to our results, of the 2194 non-recent immigrants aged between 25 and 64, the proportion of those who belonged to these job categories was lower at 44.2% (969/2194) as compared with 58.7% (1025/1745) among the recent immigrants.

On the other hand, screening opportunities for working-age immigrants who are unemployed, self-employed or homeworkers, including 36% (790/2194) of non-recent immigrants aged between 25 and 64, are limited to ad hoc TB screening campaigns organized by local authorities and nongovernmental organizations for the general community and some specifically targeting foreign residents. Although reports are limited, the take-up of community-based screenings by foreign residents appears to be low due to various barriers including language, geographical distance, lack of information and economic difficulties. (*13*)

Analysis of the TB surveillance data indicates that the proportion of sputum smear-positive PTB cases detected via routine screening has constantly been smaller than that of those detected in hospitals while being hospitalized for other diseases or while seeing a doctor for other diseases (25.6% vs 62.1% in 2015). (*14*) Another study has similarly reported smaller proportions of positive sputum smears and cavity disease among those patients with professions that are more likely to be detected via routine workplace screening. (*15*) This suggests the possibility that non-recent immigrants, who have fewer opportunities for routine school and workplace screening, are more likely to be detected with a progressed disease and thereby are at a higher risk of infecting others.

Increasing the opportunities for and improving the take-up rate of community-based screenings for non-recent immigrants may not only contribute to early detection of TB, prevention of secondary infection and better treatment outcome but also may provide improved general health, especially for the elderly immigrants. Further studies are needed to assess the cost–effectiveness of possible different routine screening programmes for those who are not eligible for workplace and school-based screenings.

### Limitations

The study is not without limitations. First, as this was a cross-sectional study in design, we merely compared the characteristics between non-recent and recent immigrants, and we could not determine whether the status of being a non-recent immigrant was a risk factor for TB. Second, the timing of entry was only dichotomized in our analysis – within five years and more than five years. This was due to the fact that the year of entry was collected only after 2012; before that, the timing of entry to Japan had only been collected as within five years, more than five years or year of entry unknown. As we had placed a greater emphasis on increasing the number of cases for analysis, the study period was set from 2007. Nonetheless, a recently published report suggests that of the non-recent immigrants approximately 40% entered between five and nine years and 60% more than 10 years before being diagnosed with TB. (*8*) Third, the general TB screening policy has undergone some changes in the recent years – for example, since 2010, a chest X-ray is no longer mandatory in the annual routine workplace screening for those aged 40 and below. The extent to which such a change affects the epidemiology of foreign-born TB remains to be investigated.

## Conclusions

The demographic and socioeconomic background of pulmonary TB among non-recent immigrants are distinctively different from that of recent immigrants. The former, being more integrated into the Japanese society, may be more invisible and hard to reach than recent immigrants. Despite being integrated, they often fail to benefit from TB programmes targeting the obvious foreign-born population in Japan such as workplace and school-based TB screening. An integrated approach, including a community-based comprehensive health check, may be necessary as part of the greater effort to control TB among the foreign-born population in Japan.
